# Tangeretin-Assisted Platinum Nanoparticles Enhance the Apoptotic Properties of Doxorubicin: Combination Therapy for Osteosarcoma Treatment

**DOI:** 10.3390/nano9081089

**Published:** 2019-07-29

**Authors:** Sangiliyandi Gurunathan, Muniyandi Jeyaraj, Min-Hee Kang, Jin-Hoi Kim

**Affiliations:** Department of Stem Cell and Regenerative Biotechnology, Konkuk University, Seoul 05029, Korea

**Keywords:** platinum nanoparticles, doxorubicin, oxidative stress, cytotoxicity, genotoxicity, apoptosis, DNA damage

## Abstract

Osteosarcoma (OS) is the most common type of cancer and the most frequent malignant bone tumor in childhood and adolescence. Nanomedicine has become an indispensable field in biomedical and clinical research, with nanoparticles (NPs) promising to increase the therapeutic efficacy of anticancer drugs. Doxorubicin (DOX) is a commonly used chemotherapeutic drug against OS; however, it causes severe side effects that restrict its clinical applications. Here, we investigated whether combining platinum NPs (PtNPs) and DOX could increase their anticancer activity in human bone OS epithelial cells (U2OS). PtNPs with nontoxic, effective, thermally stable, and thermoplasmonic properties were synthesized and characterized using tangeretin. We examined the combined effects of PtNPs and DOX on cell viability, proliferation, and morphology, reactive oxygen species (ROS) generation, lipid peroxidation, nitric oxide, protein carbonyl content, antioxidants, mitochondrial membrane potential (MMP), adenosine tri phosphate (ATP) level, apoptotic and antiapoptotic gene expression, oxidative stress-induced DNA damage, and DNA repair genes. PtNPs and DOX significantly inhibited U2OS viability and proliferation in a dose-dependent manner, increasing lactate dehydrogenase leakage, ROS generation, and malondialdehyde, nitric oxide, and carbonylated protein levels. Mitochondrial dysfunction was confirmed by reduced MMP, decreased ATP levels, and upregulated apoptotic/downregulated antiapoptotic gene expression. Oxidative stress was a major cause of cytotoxicity and genotoxicity, confirmed by decreased levels of various antioxidants. Furthermore, PtNPs and DOX increased 8-oxo-dG and 8-oxo-G levels and induced DNA damage and repair gene expression. Combination of cisplatin and DOX potentially induce apoptosis comparable to PtNPs and DOX. To the best of our knowledge, this is the first report to describe the combined effects of PtNPs and DOX in OS.

## 1. Introduction

Osteosarcoma (OS) is a malignant primary bone tumor commonly observed worldwide in both children and adults, accounting for 60% of primary bone cancers diagnosed under the age of 20 [1]. OS may occur at any age and is thought to occur more often in males than females [2]. The most common, currently-approved treatments for OS are chemotherapy and surgery [3,4]. Neoadjuvant and adjuvant chemotherapy combined with surgery increase the five-year survival rate compared to surgery alone [3,4]; however, patients with advanced stages of OS exhibit low survival rates [1]. The success rate of surgery alone against localized OS ranges from 15–20%, yet increases to approximately 70% when combined with chemotherapy [5]. There are currently no effective therapies for patients who relapse or experience undesired side effects from chemotherapy. Although there are several primary and adjuvant chemotherapeutic agents used to treat OS, doxorubicin (DOX), cisplatin, and methotrexate are the most common; therefore, discovering and developing novel biocompatible agents with less toxic side effects to improve the survival rates of patients is highly important in the field of nanomedicine.

Cancer therapies based on a single drug are ineffective due to the complex microenvironment of cancer cells and drug resistance mechanisms [6]. Combination therapies using nanoparticles (NPs) and chemotherapeutic agents appear to be viable, effective, and exhibit fewer toxic effects. Biocompatible NPs have been used for drug delivery and as cytotoxic agents since they can easily enter cells and tissues due their unique size, surface area, solubility, and bioavailability [7]. Recently, combination therapy has been considered a promising strategy for improving therapeutic efficiency and minimizing side effects [8], since the combination of two or more chemotherapeutic drugs can synergistically suppress cancer cells via two different molecular pathways to delay cancer cell adaptation and thereby reduce cancer cell mutations [9].

Nanotechnology-based cancer therapy is a groundbreaking strategy that has changed the landscape of pharmacotherapy. Nanomaterials (NMs) can improve pharmacokinetics (PKs) and site-specific delivery, have provided a useful delivery tool for combination therapy [10], facilitate various NP functions including solubility and stability, maintain the in vivo fate and dose ratio of the combined drugs, preferentially accumulate at the tumor site via the enhanced permeability and retention (EPR) effect, exhibit active targeting, improve therapeutic efficacy, are resistant to drug efflux mediated by multidrug resistance (MDR) transporters, and allow drug concentrations to be easily altered within organelles [11,12]. NPs (such as silver NPs (AgNPs)) can inhibit the proliferation of neoplasm cells and display synergistic effects with the delivered drugs [12,13,14,15].

Given the unique advantages of NPs and their promising applications in anticancer therapy, it is necessary to develop combination strategies for overcoming MDR by rationally designing optimal combined formulations of NPs with anticancer drugs. In particular, more biomolecule-mediated NPs are required for cancer therapy. Recently, Zhao et al. [16] developed an all-in-one NP consisting of multiple drugs, such as 10-hydroxycamptothecin (HCPT) and DOX, with enhanced synergistic antiproliferation efficiency against drug-resistant cancer cells. Combinations of drugs and NPs could be a synergistic approach that provides greater therapeutic effects than single-drug treatments. Furthermore, targeting multiple signaling pathways could be a new method of cancer treatment [17]. Efflux pump inhibitors, proapoptotic compounds, and siRNAs have been used as therapeutic agents for combination therapy, with NP-mediated targeted therapy providing an innovative and promising alternative to conventional small-molecule chemotherapeutics [18]. Platinum NPs (PtNPs) are nontoxic, effective, thermally stable, and have thermoplasmonic properties. In addition, PtNPs are synthesized from biomolecules in a process free from environmentally-damaging chemicals and health hazards. We selected DOX, a common clinical chemotherapeutic drug, as the chemotherapeutic agent for the combination therapy [16,19]. The advantage of using a combination of NPs and chemotherapeutic drugs that act via independent pathways is that the two molecules can achieve synergistic therapeutic effects, reducing drug resistance and side effects. Recently, we reported that the combination of AgNPs and salinomycin enhances apoptosis and autophagy in human ovarian cancer cells [20], whilst AgNPs may increase the apoptotic effects of gemcitabine in human ovarian cancer cells [21]. Another interesting study suggested that the combination of palladium NPs (PdNPs) and tubastatin-A potentiates apoptosis in human breast cancer cells by altering various cellular pathways [21]. AgNPs and the histone deacetylase inhibitor MS-375 were shown to induce mitochondrial dysfunction, autophagosome accumulation, caspase 9/3 activation, and the up- and downregulation of pro- and antiapoptotic genes in human alveolar basal epithelial cells [22].

To overcome the undesirable side effects associated with using high concentrations of chemotherapeutic agents, it is necessary to use the minimum dose required to achieve their cytotoxic, genotoxic, and apoptotic activities. We hypothesized that combining PtNPs with DOX, a potent chemotherapeutic agent, would be more effective than either single agent alone against OS cells. This study was designed to evaluate the combined effects of PtNPs and DOX in human bone OS epithelial cells (U2OS).

## 2. Materials and Methods

### 2.1. Cell Lines and Agents

Human U2OS cells (Korean Cell Line Bank, Seoul, Korea) were cultured in Dulbecco’s modified Eagle medium (Sigma-Aldrich, St. Louis, MO, USA) containing 10% fetal bovine serum and penicillin–streptomycin in 75 cm^2^ tissue culture flasks (Corning, NY, USA) at 37 °C in the presence of 5% CO_2_ at 95% relative humidity. DOX, dried and hydrated hexachloroplantinic acid (H_2_PtCl_6_·6H_2_O), cisplatin (CIS), and tangeretin were purchased from Sigma-Aldrich. Penicillin–streptomycin, trypsin–EDTA, DMEM, fetal bovine serum (FBS), and antibiotic-antimycotic reagents were obtained from Life Technologies/Gibco (Grand Island, NY, USA). The in vitro toxicology assay kit and the reagent kits for the measurement of malondialdehyde (MDA), protein carbonyl content, and antioxidants were purchased from Sigma-Aldrich. All other chemicals were purchased from Sigma-Aldrich unless otherwise stated.

### 2.2. PtNPs Synthesis and Characterization 

PtNPs were synthesized by reduction of PtCl_6_^2−^ ions into PtNPs by mixing 1 mg/mL tangeretin with 45 mL of 1 mM aqueous H_2_PtCl_6_·6H_2_O and incubating the mixture at 100 °C on a hotplate for 1 h in a sealed flask to avoid evaporation, since temperature catalyzes the reduction process. For the control experiments, identical amounts of Pt solution and tangeretin were maintained separately under the same reaction conditions. The reduced Pt solution was sonicated for 10 min to separate Pt nanomaterials from the biomolecules and filtered with a 0.2 µm syringe filter. The reduced Pt was purified by repeated centrifugation at 5000 rpm for 30 min and the pellets were washed with distilled water to remove impurities. Purified PtNPs were characterized by UV-visible (UV-vis) spectroscopy. UV-vis spectra were obtained using a Biochrom WPA Biowave II UV/Visible Spectrophotometer (Biochrom, Cambridge, UK). Particle size and zeta potential were measured by Zetasizer Nano ZS90 (Malvern Instruments, Limited, Malvern, UK). X-ray diffraction (XRD) analysis was performed in a Bruker D8 DISCOVER X-ray diffractometer (Bruker AXS GmbH, Karlsruhe, Germany). Dry PtNPs powder was suspended in KBr, and Fourier transform infrared (FTIR) spectroscopy was performed using a Spectrum GX spectrometer (Perkin Elmer Inc., Waltham, MA, USA) within the range of 500–4000 cm^−1^. Transmission electron microscopy (TEM) using a JEM-1200EX microscope (JEOL Ltd., Akishima-shi, Japan) was performed to determine the size and morphology of AgNPs. TEM images of AgNPs were obtained at an accelerating voltage of 300 kV [23].

### 2.3. Cell Culture Conditions

U2OS cells were cultured in DMEM supplemented with 10% FBS, 100 U/mL penicillin, and 0.1 mg/mL streptomycin (Sigma-Aldrich). The cells were usually subcultured twice a week with 1 × 10^6^ viable cells/mL and incubated at 37 °C in a 5% CO_2_ atmosphere. The medium was replaced the next day with fresh medium and the cells were incubated for 24 h prior to PtNPs or DOX exposure. Experiments were performed in 96-, 24-, and 12-well plates and 100 mm cell culture dishes, as required. Cells were treated with various concentrations of PtNPs, DOX, and CIS, or a combination of PtNPs (10 μg/mL) and DOX (1.0 μg/mL) or combination of DOX (1.0 μg/mL) and CIS (5.0 μg/mL) for 24 h. Cells cultured without PtNPs or DOX or CIS were used as the control.

### 2.4. Cell Viability Assay

Cell viability was measured using cell counting kit-8 (CCK-8; CK04-01, Dojindo Laboratories, Kumamoto, Japan). Briefly, U2OS cells were plated in 96-well flat-bottomed culture plates containing various concentrations of PtNPs, DOX, or PtNPs and DOX. After culturing for 24 h at 37 °C in a humidified 5% CO_2_ incubator, CCK-8 solution (10 μL) was added to each well and the plate was incubated for 2 h at 37 °C. The absorbance was measured at 450 nm using a microplate reader (Multiskan FC; Thermo Fisher Scientific Inc., Waltham, MA, USA).

### 2.5. BrdU Cell Proliferation Assay

Cell proliferation was determined according to the manufacturer’s instructions (Roche). Cells were incubated with various concentrations of PtNPs for 24 h, with the BrdU labeling solution added to the culture medium 2 h before the end of the incubation. The cells were fixed and the level of incorporated BrdU was determined using a BrdU enzyme-linked immunosorbent assay (ELISA) kit (Roche) according to the manufacturer’s instructions. The proliferation of the untreated cells at 0 h was considered 100%.

### 2.6. Determination of PtNPs Concentration Using Inductively Coupled Plasma Mass Spectrometry (ICP-MS) 

ICP-MS (Santa Clara, CA, USA) was performed to measure the Pt contents in the PtNPs exposed cells. Cells were treated with 15 µg/mL of PtNPs for 24 h, and then cells were collected by Trypsin/EDTA treatment. PtNPs was measured in the cells by ICP-MS. Briefly, 1 × 10^6^ SH-SY5Y cells were cultured on the each six-well cell culture plate, treated with PtNPs. After treating PtNPs, the cells were collected by trypsin/EDTA, and PtNPs were measured.

### 2.7. Assessment of Membrane Integrity 

The membrane integrity of U2OS cells was evaluated using a lactate dehydrogenase (LDH) cytotoxicity detection kit. Briefly, cells were exposed to various concentrations of PtNPs for 24 h, 100 μL of cell-free supernatant from each well was transferred in triplicate into the wells of a 96-well plate, and 100 μL of LDH reaction mixture was added to each well. After 3 h of incubation under standard conditions, the optical density of the final solution was determined at a wavelength of 490 nm using a microplate reader.

### 2.8. Cell Mortality Assay

Cell mortality was evaluated using the trypan blue assay, as described previously [23]. U2OS cells were plated in six-well plates (1 × 10^5^ cells per well) and incubated for 24 h with various concentrations of PtNPs. Cells cultured without PtNPs, DOX, or PtNPs and DOX were used as controls. After 24 h, the cells were detached using 300 µL trypsin–EDTA solution, both adherent and suspended cells were collected, and the mixture of the supernatant and detached cells was centrifuged at 1200 rpm for 5 min. The pellet was mixed with 700 µL trypan blue solution, dispersed, stained for 5 min, and then cells were counted using a cytometer. Viable cells were unstained and dead cells were stained blue. Three independent experiments were performed in triplicate. The mean and standard deviation were calculated, with cell proliferation expressed as the percentage of viable cells relative to the appropriate control.

### 2.9. Cell Morphology

U2OS cells were plated in six-well plates (2 × 10^5^ cells per well) and incubated with PtNPs (10 μg/mL), DOX (1 μg/mL), CIS (5 μg/mL), or a combination of PtNPs (10 μg/mL) and DOX (1 μg/mL) or combination of DOX (1 μg/mL) and CIS (5 μg/mL) for 24 h. Cells cultured without PtNPs or DOX were used as the control. The cell morphology was analyzed using an optical microscope at 24 h post-treatment. The morphology of the cells was examined with an OLYMPUS IX71 microscope (Tokyo, Japan) using the appropriate filter sets.

### 2.10. Determination of ROS, MDA, Nitric Oxide (NO), and Carbonylated Protein Levels 

ROS were estimated as described previously [23]. U2OS cells were seeded in 24-well plates (5 × 10^4^ cells per well) and cultured for 24 h. After washing twice with phosphate-buffered saline (PBS), fresh medium containing PtNPs (10 μg/mL), DOX (1 μg/mL), CIS (5 μg/mL), or a combination of PtNPs (10 μg/mL) and DOX (1 μg/mL) or combination of DOX (1 μg/mL) and CIS (5 μg/mL) for 24 h. The cells were supplemented with 20 μM DCFH2-DA and incubated for a further 30 min at 37 °C. The cells were rinsed with PBS, 2 mL PBS was added to each well, and the fluorescence intensity was determined using a Gemini EM spectrofluorometer (Molecular Devices, Sunnyvale, CA, USA) at an excitation wavelength of 485 nm and an emission wavelength of 530 nm.

The expression level of oxidative stress markers was measured as described previously [23]. MDA level was measured according to the method described earlier [23]. Briefly, the U2OS cells were seeded into six-well microplates at 2.0 × 10^6^ cells per well. The cells were treated with PtNPs (10 μg/mL), DOX (1 μg/mL), CIS (5 μg/mL), or a combination of PtNPs (10 μg/mL) and DOX (1 μg/mL) or combination of DOX (1 μg/mL) and CIS (5 μg/mL) for 24 h. After incubation, the cells were harvested and washed twice with an ice-cold PBS solution. The cells were collected and disrupted by ultrasonication for 5 min on ice. The cell extract (100 μL) was used to detect MDA according to the procedure recommended by the manufacturer of the MDA assay kit. To measure the MDA, the cells were washed twice with PBS, scraped from the plates into ice-cold PBS (0.1 M, containing 0.05 mM EDTA), and homogenized. The homogenate was centrifuged at 4 °C at 10,000 rpm for 15 min, and the resulting supernatant was stored at 70 °C until analyzed. Protein concentration was determined using the Bradford method with bovine serum albumin (BSA) as a reference standard. The concentration of MDA was measured on a microplate reader at a wavelength of 530 nm. The protein concentration was determined using the Bio-Rad protein assay kit (Bio-Rad, Hercules, CA, USA). Nitric oxide (NO) production was measured from the cells treated with PtNPs (10 μg/mL), DOX (1 μg/mL), CIS (5 μg/mL), or a combination of PtNPs (10 μg/mL) and DOX (1 μg/mL) or combination of DOX (1 μg/mL) and CIS (5 μg/mL) for 24 h and the amount of NO was quantified spectrophotometrically using the Griess reagent. The absorbance was measured at 540 nm and the nitrite concentration was determined using a calibration curve prepared with sodium nitrite as the standard [23]. The cells were harvested in chilled PBS, by scraping and washing twice with 1× PBS at 4 °C for 6 min at 1500 rpm. The cell pellet was sonicated at 15 W for 10 s (three cycles) to obtain the cell lysate. The resulting supernatant was stored at −70 °C until analyzed. Carbonylated protein content was measured as described previously [23]. Protein Carbonyl Content was measured in U2OS cells after 24 h exposure to PtNPs (10 μg/mL), DOX (1 μg/mL), CIS (5 μg/mL), or a combination of PtNPs (10 μg/mL) and DOX (1 μg/mL) or combination of DOX (1 μg/mL) and CIS (5 μg/mL). Estimation was measured by dissolving samples in dH_2_O and centrifuge to spin down any insoluble. Dilute samples with dH_2_O to approximately 10 mg/mL protein and the samples were used approximately 100 μL of sample containing approximately 0.5–2 mg protein per assay.

### 2.11. Measurement of Antioxidative Marker Levels

The expression levels of oxidative and antioxidative stress markers were measured as described previously [23]. PtNPs (10 μg/mL), DOX (1 μg/mL), CIS (5 μg/mL), or a combination of PtNPs (10 μg/mL) and DOX (1 μg/mL) or combination of DOX (1 μg/mL) and CIS (5 μg/mL) for 24 h. After the treatment, the cells were washed twice with PBS, scraped from the plates into ice-cold PBS (0.1 M, containing 0.05 mM EDTA), and homogenized. Further, the cell pellet was sonicated three times at 15 W for 10 s to obtain the cell lysate. The resulting supernatant was stored at −70 °C for further analysis. The homogenate was centrifuged at 4 °C at 10,000 rpm for 15 min, and the resulting supernatant was stored at 70 °C until analyzed. Protein concentration was determined using the Bradford method with bovine serum albumin (BSA) as a reference standard. The levels of antioxidative stress markers, reduced glutathione (GSH), thioredoxin (TRX), catalase (CAT), superoxide dismutase (SOD), glutathione peroxidase (GPx), and glutathione S-transferase (GST) were determined according to the manufacturer’s instructions.

Glutathione reductase activity was measured by monitoring the rate of NADPH oxidation 1 × 10^6^ cells were lysed in 50 μL of 100 mM potassium phosphate buffer (pH 7.5) containing 1 mM EDTA and 1% Triton X-100. Cell lysate was mixed with 50 μL of 4 mM GSSG solution and 100 μL of 200 μM NADPH solution (Sigma-Aldrich). Oxidation of NADPH was monitored spectrophotometrically in kinetic mode for 5 min at 340 nm. Glutathione reductase activity was proportional to the rate of absorbance decrease.

Glutathione peroxidase activity was measured indirectly by monitoring NADPH consumption in a coupled reaction with glutathione reductase (Smith and Levander, 2002). 1 × 10^6^ cells were lysed in 50 μL of 50 mM Tris-HCl buffer (pH 8.0) containing 0.5 mM EDTA and 0.1% Triton X-100. Cell lysate was mixed with 50 μL of solution containing 1 mM NADPH, 8.5 mM GSH, 2 U/mL GR (Sigma-Aldrich). Reaction was initiated by adding 100 μL of 0.6 mM tert-butyl hydroperoxide (Sigma-Aldrich) solution. Oxidation of NADPH, coupled with oxidized glutathione reduction, was monitored spectrophotometrically in kinetic mode for 5 min at 340 nm. Glutathione peroxidase activity was proportional to the rate of absorbance decrease.

Thioredoxin reductase activity was measured by monitoring the conversion of DTNB to TNB by reduced thiols. A total of 1 × 10^6^ cells was lysed in 50 μL of 100 mM potassium phosphate buffer (pH 7.5) containing 1 mM EDTA and 1% Triton X-100. Another aliquot of the same cell sample was lysed in the same lysis buffer, but containing additionally 3 μM auranofin as thioredoxin reductase inhibitor. Cell lysate was mixed with 100 μL of solution containing 5 mM DTNB and 250 μM NADPH. Absorbance was monitored spectrophotometrically in kinetic mode for 5 min at 405 nm.

Catalase activity was assayed by monitoring the rate of removal of exogenously added hydrogen peroxide in a colorimetric reaction with Trinder’s reagent. 1 × 10^6^ cells were lysed in 50 μL of 50 mM potassium phosphate buffer (pH 7.0) containing 0.1% Triton X-100, and incubated for 3 minutes with 50 mM hydrogen peroxide. The catalase reaction was stopped by adding sodium azide to 15 mM final concentration and the remaining hydrogen peroxide was assayed in a reaction with Trinder’s reagent (final concentrations 0.225 mM 4-aminoantipyrine, 1.8 mM 3,5-dichloro-2-hydroxybenzenosulfonic acid, 0.675 U/mL horseradish peroxidase; Sigma-Aldrich) for 30 min. Absorbance was measured at 520 nm. Catalase activity was proportional to the difference in absorbance between a control sample (without cell lysate) and the assayed sample.

Superoxide dismutase activity was assayed by monitoring the rate of removal of exogenously added superoxide in a colorimetric reaction with nitro blue tetrazolium. In total, 1 × 10^6^ cells were lysed in 50 μL of 10 mM potassium phosphate buffer (pH 7.4) containing 0.1 mM EDTA and 10 μM diphenylene iodonium for 1 min in an ultrasound bath. Cell lysate was extracted with ethanol/chloroform at 20% and 11% final concentrations, respectively, to remove proteins other than SOD which might have dismutase activity. Fifty microliters of aqueous phase was mixed with 100 μL of solution containing 160 μM NADH and 100 μM nitro blue tetrazolium (Sigma-Aldrich). 50 μL of 13 μM phenazine methosulfate (Sigma-Aldrich) solution was added and the change in absorbance was monitored in kinetic mode for 5 min at 560 nm. Superoxide dismutase activity was proportional to the difference in absorbance increase rate between a control sample (without cell lysate) and the assayed sample.

GSTs activity was assayed spectrophotometrically at 25 °C with reduced glutathione (GSH) and 1-chloro-2, 4-dinitrobenzene (CDNB) as substrates. This was done by watching an increase in absorbance at 340 nm. For each assay, one mL of assay cocktail (980 μL PBS pH 6.5, 10 μL of 100 mM CDNB and 10 µL of 100 mM GSH) was prepared, then 100 µL of cocktail was removed and the remaining 900 µL was placed into a 1.5 mL cuvette. As a blank, the absorbance of a cuvette with 100 μL PBS and 900 μL of cocktail was measured at 340 nm, every 1 min, for 3 min. To the test cuvette, 100 μL of sample was added to 900 μL cocktail, mixed, and the absorbance was measured at 340 nm.

### 2.12. Measurement of 8-oxo-7,8-dihydro-2′-deoxyguanosine (8-oxo-dG) and 8-oxo-G Levels

U2OS cells were treated with PtNPs (10 μg/mL), DOX (1 μg/mL), CIS (5 μg/mL), or a combination of PtNPs (10 μg/mL) and DOX (1 μg/mL) or combination of DOX (1 μg/mL) and CIS (5 μg/mL) for 24 h. 8-oxo-dG and 8-oxo-G contents were determined as described previously [23,24,25] and as per the manufacturer’s instructions (Trevigen, Gaithersburg, MD, USA). First, we prepared an appropriate number of 8-OHdG precoated well strips and then equilibrated with all reagents at room temperature; then, HRP-conjugated 8-OHdG antibody was added to either standards or samples in the appropriate wells. After incubation for 60 min, all unbound reagents were removed. Finally, the absorbance was measured with an ELISA plate reader set at 450 nm.

### 2.13. Reverse Transcription-Quantitative Polymerase Chain Reaction (RT-qPCR)

Total RNA was extracted from U2OS cells treated with PtNPs (10 μg/mL), DOX (1 μg/mL), CIS (5 μg/mL), or a combination of PtNPs (10 μg/mL) and DOX (1 μg/mL) or combination of DOX (1 μg/mL) and CIS (5 μg/mL) for 24 h using the PicoPure RNA isolation kit (Arcturus Bioscience, Mountain View, CA, USA) according to the manufacturer’s instructions. RT-qPCR was conducted using a Vill7 device (Applied Biosystems, Foster, CA, USA) with SYBR Green as the double-stranded DNA-specific fluorescent dye (Applied Biosystems). Target gene expression levels were normalized to glyceraldehyde-3-phosphate dehydrogenase (GAPDH) expression, which was unaffected by the treatment. The PCR primer sequences are shown in Table 1.

### 2.14. Statistical Analysis

Independent experiments were repeated at least three times and data represent the mean ± standard deviation (SD) of all replicates within an individual experiment. Data were analyzed using the *t*-test, multivariate analysis, or one-way analysis of variance (ANOVA) followed by Tukey’s test for multiple comparisons to determine the significant differences between groups (*). The results are presented as mean ± standard deviation of three experiments. * *p* < 0.05 was considered significant; ** *p* < 0.01 was considered highly significant and *** *p* < 0.001 was considered very highly significant.

## 3. Results and Discussion

### 3.1. Synthesis and Ccharacterization of PtNPs Using Tangeretin

**Figure 1:**
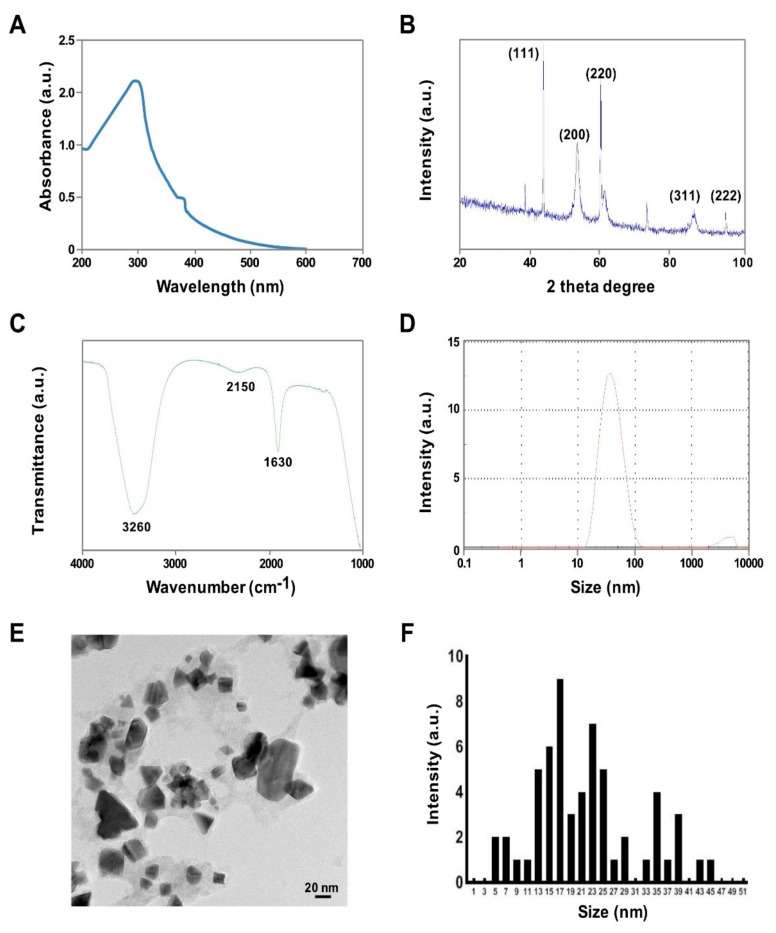
Synthesis and characterization of PtNPs using tangeretin. (**A**) Absorption spectra of tangeretin-mediated PtNP synthesis; the absorption spectra of PtNPs exhibited a strong broad peak at 304 nm, and observation of such a band is assigned to surface plasmon resonance of the particles. (**B**) X-ray diffraction patterns of PtNPs; the broad diffraction peaks of the XRD pattern at 2θ = 40.0, 47.6, 67.5, 81.8, and 88.6° were observed (**C**) FTIR spectra of PtNPs; (**D**) size distribution analysis of PtNPs by DLS; (**E**) TEM micrograph images of PtNPs; several fields were photographed and used to determine the diameter of AgNPs using TEM; and (**F**) corresponding particle size distribution histograms. At least three independent experiments were performed obtaining reproducible results and data from representative experiments are shown.

PtNPs synthesis was carried out using tangeretin and H_2_PtCl_6_·H_2_O, which is an ideal metal precursor due to its good solubility in polar solvents [26]. Typically, the color of the H_2_PtCl_6_ solution is yellow, with the color of the mixed solution changing quickly from yellow to dark brown, suggesting that Pt ions have been completely reduced to PtNPs [23,27]. UV–Vis spectroscopy is an important and valuable technique for determining the formation and stability of metal NPs in an aqueous solution [28]. Figure 1A shows the UV–Vis spectra of the green synthesized PtNPs, depicting a single maximum absorption band at 304 nm which indicates the complete reduction of Pt ions [29]. Similarly, Al-Radadi et al. [30] reported that the plasmon resonance values of PtNPs produced using Ajwa and Barni dates were 321 and 329 nm, respectively; however, the values depended on the size and shape of the PtNPs [31,32,33]. As a reducing and stabilizing agent, tangeretin produces less agglomerated PtNPs with heterogeneous shapes, including some identifiable cubic and tetrahedral particles. Michel et al. [34] synthesized heterogeneous PtNPs by reducing aqueous [PtCl_4_]^2−^ solutions at room temperature with H_2_ as a reducing agent and either glycolate or tartrate ions as shape-directing agents. Tetrahedral NPs are catalytically active, the most sensitive to shape changes, and have sharp corners and edges with the least surface energy that can be easily reconstructed following chemical perturbation [35].

Next, we determined the phase purity and crystal structure of the synthesized NPs by X-ray diffraction. As shown in Figure 1B, the broad reflections of the synthesized powder indicate that it had a nanocrystalline nature [36]. The broad diffraction peaks of the XRD pattern at 2θ = 40.0, 47.6, 67.5, 81.8, and 88.6° correspond to reflections (111), (200), (220), (311), and (222), respectively (Figure 1B), consistent with the face centered cubic (fcc) structure assigned to Pt (JCPDS Card 04-0802) and demonstrating the presence of crystalline Pt. The synthesized particles showed significant agreement with PtNPs prepared at 190 °C using water as solvent [36]. FTIR spectroscopy was used to determine which biomolecules were responsible for PtNPs synthesis. The FTIR spectra exhibited characteristic bands at 1630 and 3260 cm^−1^, corresponding with the bending and stretching vibrations of the amide I bond, respectively (Figure 1C). The peak at 3260 cm^−1^ is associated with O–H stretching (intramolecular hydrogen bonded OH), whilst the band at 2150 cm^−1^ may indicate the presence of an alkyne group. These findings are supported by previous studies reporting that biomolecules are responsible for Pt ion reduction [23,30,37].

DLS measurements were carried out to determine the average size and size distribution of the prepared PtNPs. The DLS measurements indicated that the prepared PtNPs exhibited an average size of 30 nm (Figure 1D), which was slightly larger than the size obtained from TEM-based analysis but in good agreement with published results [23]. Furthermore, DLS showed that the prepared NPs were not agglomerated, which is highly important for toxicity evaluation. Zeta potential measurement gives an idea of the surface charge associated with the particles. In this experiment, we measured zeta potential of tangeretin stabilized PtNPs. The particle charge of nanoparticles determines physical stability of mixtures and suspensions. The zeta potential of prepared PtNPs exhibited −45.8 mV, which are considered to be more stable due to more negative charge of the particles and also it suggest that tangeretin biomolecules significantly increase the stability of the bioreduced PtNPs.

The size and shape of the PtNPs was determined by TEM. TEM micrographs revealed that the size of the particles was 10–22 nm (Figure 1E,F) and confirmed that the synthesized particles were spherical in shape. Uniform spherical particles have also been obtained using microwave irradiation [38] and agro-industrial waste *Punica granatum* peel extract [39]. The size of the PtNPs obtained using tangeretin was similar to those derived from *Punica granatum* crust, which had an average size of 20 nm [40]. In contrast, crystalline and irregular rod-shaped NPs were obtained using dried leaf powder from *Anacardium occidentale* [29]. Collectively, these results show that PtNPs synthesized from tangeretin are free from environmentally hazardous substances, nontoxic, environmentally friendly, and a suitable size for biomedical applications.

### 3.2. Dose-Dependent Effect of PtNPs, DOX, and CIS on U2OS Cell Viability

**Figure 2:**
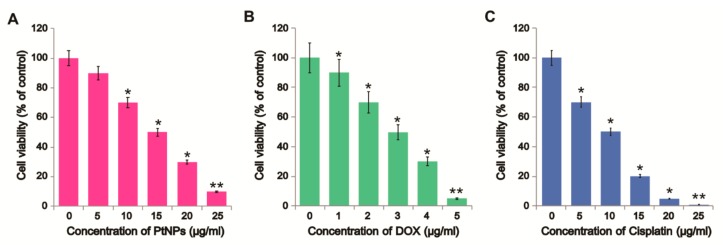
Dose-dependent effect of PtNPs, DOX, and CIS on U2OS cell viability. U2OS cell viability determined after exposure to different concentrations of (**A**) PtNPs (5–25 µg/mL), (**B**) DOX (1–5 µg/mL), or (**C**) CIS (5–25 µg/mL) for 24 h. Results are expressed as the mean ± standard deviation of three independent experiments. Differences between the treated and control groups were measured using Student’s *t*-test and statistically significant differences are indicated by * (*p* < 0.05). * *p* < 0.05 was considered significant; ** *p* < 0.01 was considered highly significant and *** *p* < 0.001 was considered very highly significant.

DOX, one of the most effective anticancer drugs, was selected for this study since it can cause DNA damage, inhibit DNA replication, and consequently cause apoptosis [41]; however, its toxicity is not restricted to cancer cells as it can also cause adverse side effects in healthy cells [41]. We first assessed the effects of PtNPs and DOX on U2OS cell viability. U2OS cells were treated with various concentrations of PtNPs (5–25 μg/mL) or DOX (1–5 μg/mL) or cisplatin (5–25 μg/mL) for 24 h. PtNPs decreased cell viability in a dose-dependent manner with significant decreases observed at high concentrations with an IC50 value of 15 μg/mL, whereas DOX suppressed cell viability at 1 μg/mL with an IC50 value of 3 μg/mL (Figure 2A,B). Similarly, as a positive control we used cisplatin to determine the effect on cell viability. The results suggest that cisplatin dose-dependently decrease the viability of U2OS cells (Figure 2C). The IC50 value of CIS found to be 10 μg/mL. Sack et al. [41] reported that DOX exerted strong cytotoxic effects in A375 cells at much lower concentrations and after shorter incubation times than cerium oxide NPs (CeO_2_NPs); however, combining CeO_2_NPs with DOX inhibited cell viability more strongly [41]. Recently, we reported that apigenin-functionalized PtNPs dose-dependently reduced the viability of human monocytic THP-1 cells, whilst vanillin-functionalized graphene oxide PtNPs (GOPtNPs) induced cytotoxicity in human prostate cancer cells (LNCaP) by reducing cell viability and proliferation and increasing oxidative stress. Ji et al. [42] reported that OS cell viability was reduced by the upregulation of caspase 3 activity in a time- and dose-dependent manner, whilst DOX has been shown to reduce cell viability in a dose- and time-dependent manner in MCF-10F, MCF-7m, and MDA-MB-231 cells [43]. Collectively, these findings suggest that biomolecule-functionalized PtNPs can exhibit cytotoxicity and that U2OS cells are sensitive to PtNPs and DOX.

### 3.3. Dose-Dependent Effect of PtNPs, DOX, and CIS on U2OS Cell Proliferation

**Figure 3:**
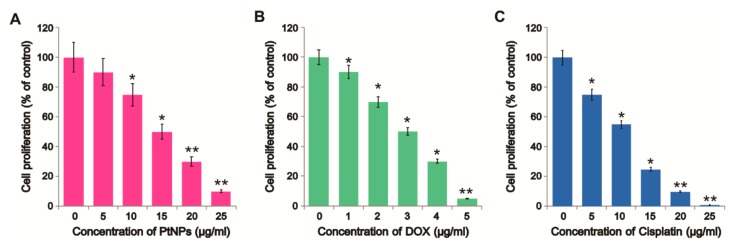
Dose-dependent effect of PtNPs, DOX, and CIS on U2OS cell proliferation. U2OS cell proliferation was determined after exposure to different concentrations of (**A**) PtNPs (5–25 µg/mL) or (**B**) DOX (1–5 µg/mL) or (**C**) CIS (5–25 µg/mL) for 24 h. Results are expressed as the mean ± standard deviation of three independent experiments. Differences between the treated and control groups were measured using Student’s *t*-test and statistically significant differences are indicated by * (*p* < 0.05). The results are presented as mean ± standard deviation of three experiments. * *p* < 0.05 was considered significant; ** *p* < 0.01 was considered highly significant and *** *p* < 0.001 was considered very highly significant.

To further investigate the antiproliferative effects of PtNPs and DOX in OS cells, we treated U2OS cells with varying concentrations of PtNPs (5–25 µg/mL) and DOX (1–5 µg/mL) and performed BrdU proliferation assays. As expected, PtNPs, DOX, and CIS significantly and dose-dependently inhibited U2OS cell proliferation (Figure 3A–C). The results suggest that cisplatin dose-dependently decrease the proliferation. A previous study showed that different concentrations of tea polyphenol-functionalized PtNPs reduced the viability and proliferation of cervical cancer cells (SiHa) and altered their nuclear morphology and cell cycle distribution [44]. Furthermore, PtNPs biologically synthesized using *Punica granatum* crusts inhibited MCF-7 proliferation with an IC_50_ of 17.84 μg/mL after 48 h of incubation [40]. We obtained an IC_50_ of 15 μg/mL, consistent with this previous report. Kumari et al. [45] reported that the combination of curcumin loaded selenium NPs (Se-CurNPs) and DOX and CD44-targeted DOX-loaded NPs (PSHA-DOXNPs) remarkably reduced HCT116 cell proliferation. Similarly, PtNPs were shown to dose-dependently inhibit the proliferation of human monocytic THP-1 and prostate cancer (LNCaP) cells [46], whilst Pt nanocomposite (PtNCP) beads reduced the viability of oral squamous cell carcinoma (HSC-3-M3) cells [47]. GOPtNPs significantly reduced cell viability and increased apoptosis in Colo 205 and HepG2 cells; however, no significant effects was observed in HT-29, HTC-116, SW480, MCF-7, LNCaP, or Hela B cells [48].

### 3.4. Cellular Quantification of PtNPs Using ICP-MS

To further cross-check our results, we also determined the amount of PtNPs taken up by using the well-established ICP-MS approach with the same experimental treatment conditions. SH-SY5Y cells were incubated with 15 µg/mL of PtNPs for 24 h. In total, three and six different replicates for the control and the samples incubated with PtNPs, respectively, were measured by ICP-MS. We found 2215 ± 250 PtNPs within the cells.

### 3.5. Combined Effects of PtNPs and DOX

**Figure 4:**
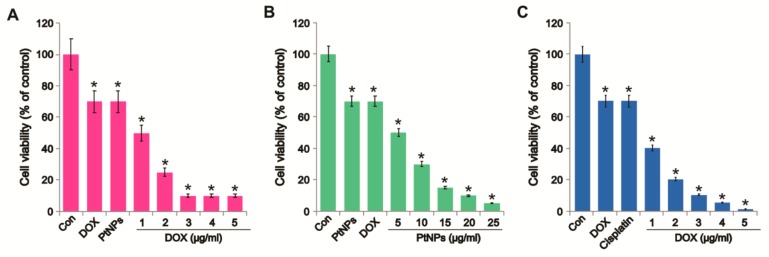
Dose-dependent effect of DOX, PtNPs, and CIS on U2OS cell viability. (**A**) U2OS cells were incubated with different DOX concentrations (1–5 μg/mL) and a fixed PtNPs concentration (10 μg/mL) for 24 h and cell viability was measured using CCK-8. (**B**) U2OS cells were incubated with different PtNPs concentrations (5–25 μg/mL) and a fixed DOX concentration (1 μg/mL) for 24 h and cell viability was measured using CCK-8. (**C**) U2OS cells were incubated with different concentrations of DOX (1–5 μg/mL) and a fixed CIS concentration (5 μg/mL) for 24 h and cell viability was measured using CCK-8. Results are expressed as the mean ± standard deviation of three independent experiments. Differences between the treated and control groups were measured using Student’s *t*-test and statistically significant differences are indicated by * (*p* < 0.05). The results are presented as mean ± standard deviation of three experiments. * *p* < 0.05 was considered significant; ** *p* < 0.01 was considered highly significant and *** *p* < 0.001 was considered very highly significant.

To determine the optimum combination concentrations for avoiding undesired toxic effects, U2OS cells were treated with different combinations of PtNPs and DOX. We examined the effect of simultaneously adding DOX (1–5 μg/mL) with a fixed concentration of PtNPs (10 μg/mL) and simultaneously adding PtNPs (5–25 μg/mL) with a fixed concentration of DOX (1 μg/mL) in U2OS cells. Increasing the concentration of PtNPs significantly reduced cell viability; however, the combination of PtNPs and DOX synergized considerably at low concentrations and reduced cell viability more than higher concentrations of PtNPs or DOX alone. This indicates that lower concentrations of PtNPs and DOX are able to produce a synergistic action and induce cell death in U2OS cells compared to higher concentrations of either alone (Figure 4A). Higher concentrations of either PtNPs or DOX were able to reduce cell viability more than lower concentrations, as expected. These results indicate that lower concentrations of PtNPs are sufficient to induce U2OS cell death. Figure 4B shows that the combination of PtNPs and DOX inhibited U2OS cells in a significant and concentration-dependent manner, whereas the PtNPs and DOX were less effective when used alone. As shown in Figure 4C, the combination of CIS and DOX inhibited U2OS cells in a significant and concentration-dependent manner, whereas the CIS and DOX were less effective when used alone. So far, no studies have described the combined effects of PtNPs and DOX in OS cancer cells. Although both PtNPs and DOX reduced cell viability individually, the effect was much more pronounced for the combined dose. A previous study reported that the combination of inorganic phosphate (Pi) and DOX efficiently inhibited the viability and proliferation of OS cells, with Pi potentiating the cytotoxic effect of DOX. DOX and curcumin coencapsulated lipid-coated polymeric NPs showed significant cytotoxic effects in human OS cell lines and exhibited antitumor effects in animal models [49]. Kumari et al. [45] reported that the combination of PSHA-DOXNPs and Se-CurNPs reduced cell viability to a greater extent than individual doses of Se-CurNPs and PSHA-DOXNPs. Furthermore, combining the autophagy inhibitor spautin-1 and DOX was shown to reduce cell survival and colony formation in canine appendicular OS cells [50]. Collectively, these findings suggest that combining PtNPs and DOX could reduce cell viability and proliferation more than either PtNPs or DOX alone. In order to avoid severe side effects and determine compatible, feasible, physiological concentrations, further combination experiments were carried out using PtNPs or DOX or CIS, with less toxic concentrations of PtNPs (10 μg/mL) and DOX (1 μg/mL) or CIS (5 μg/mL) and DOX (1 μg/mL) selected for further analysis.

The resulting combination index (CI) was then used to determine the outcome of the drug combination effect as (i) additive effect (CI = 1), (ii) synergism (CI < 1), or (iii) antagonism (CI > 1). Our results indicated that all combinations of PtNPs with DOX gave rise to CI values significantly below 1 at fa (fractional growth inhibition) = 0.5, indicating synergistic effect.

### 3.6. DOX Release Kinetics under Different Physiological Conditions 

**Figure 5:**
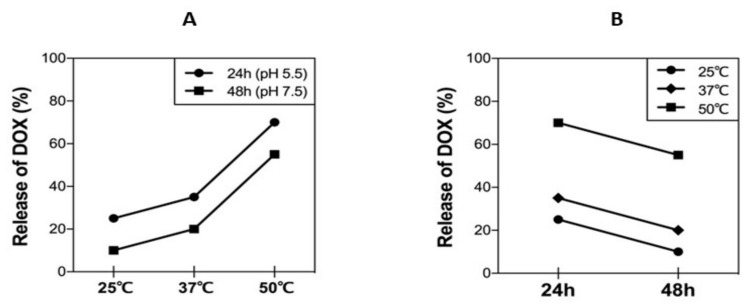
In vitro cumulative drug release profiles of DOX dispersed in PBS at 25 °C, 37 °C, and 50 °C at 24 h and 48 h in pH = 5.5 and pH = 7.5. (**A**) Temperature-dependent release kinetics of DOX; (**B**) Time-dependent release kinetics of DOX.

Drug release profile as a function of pH and temperature was carried out at different temperatures of 25, 37, and 50 °C in PBS at pH = 7.5 or 5.5. The drug release profiles of DOX under each condition show two stages: a rapid release of DOX is obtained within 24 h followed by a slower rate of release. This behavior is more pronounced at pH = 5.5 and 50 °C with 70.0% of DOX released after 24 h compared to at pH = 7.5 and 50 °C with 50.0% after 24 h, while at pH = 5.5 and 37 °C, 35.0% of DOX is released after 24 h (Figure 5A,B). The pH and the temperature greatly influence the release behavior of DOX. The minimal release of DOX is obtained at pH = 7.5 and 25 °C (followed by pH = 7.5 and 37 °C) and a more pronounced effect was observed at low pH and higher temperature, because at higher temperature, the hydrolysis kinetics are high compared to 25 °C and 37 °C. The maximal release of DOX was found at pH = 5.5 and 50 °C (Figure 5A,B), when the DOX-PtNPs were exposed to both pH and temperature stimuli. Thus, by combining both the effect of the pH and the temperature, a controlled release of the DOX can be achieved.

### 3.7. Determination of Combination Index of PtNPs and DOX

In order to determine the combination index, half-maximal inhibitory concentration (IC_50_) was calculated for each sample according to the in vitro cytotoxicity results. The synergistic effect of PtNPs and DOX was evaluated by a combination index (CI) analysis based on Chou and Talalay’s method. CI values for PtNPs and DOX were calculated using the following equation:

CI = (D)_PtNPs_/(D_X_)_PtNPs_ + (D)_DOX_/(D_X_)_DOX_

where (D)_PtNPs_ and (D)_DOX_ are the concentrations of PtNPs and DOX in the combination system at the IC_X_ value; (D_X_)_PtNPs_ and (D_X_)_DOX_ are IC_X_ value of PtNPs alone and DOX alone. CI_X_ < 1 represents synergism and CI_X_ > 1 represents antagonism. In this study, CI_50_ values were applied and the IC_50_ values were used for calculation. The resulting combination index (CI) was then used to determine the outcome of the drug combination effect as (i) additive effect (CI = 1), (ii) synergism (CI < 1), or (iii) antagonism (CI > 1). Our results indicated that all combinations of PtNPs with DOX gave rise to CI values significantly below 1 at fa (fractional growth inhibition) = 0.5, indicating a synergistic effect.

### 3.8. PtNPs and DOX Enhance LDH Leakage and Cell Death

**Figure 6:**
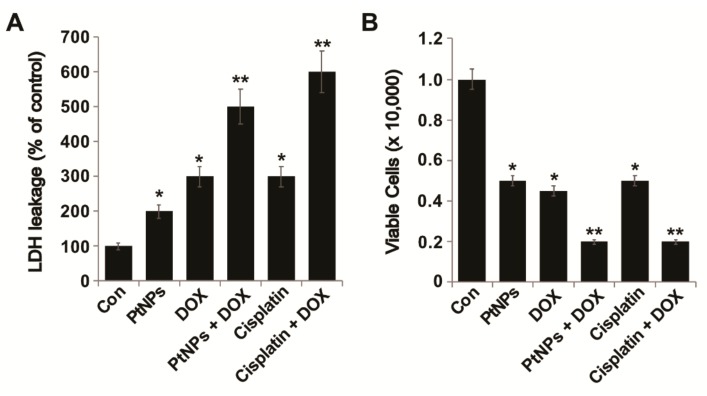
PtNPs and DOX increase LDH leakage and cell death. U2OS cells were treated with PtNPs (10 μg/mL), DOX (1 μg/mL), CIS (5 μg/mL), or a combination of PtNPs (10 μg/mL) and DOX (1 μg/mL) or combination of DOX (1 μg/mL) and CIS (5 μg/mL) for 24 h. (**A**) LDH activity was measured at 490 nm using an LDH cytotoxicity kit; (**B**) cell death was quantified using a trypan blue assay. At least three independent experiments were performed for each sample. Differences between the treated and control groups were measured using Student’s *t*-test and statistically significant differences are indicated by * (*p* < 0.05). The results are presented as mean ± standard deviation of three experiments. * *p* < 0.05 was considered significant; ** *p* < 0.01 was considered highly significant and *** *p* < 0.001 was considered very highly significant.

In order to assess membrane integrity and cytotoxicity, U2OS cells were treated with PtNPs (10 μg/mL), DOX (1 μg/mL), CIS (5 μg/mL), or a combination of PtNPs (10 μg/mL) and DOX (1 μg/mL) or combination of DOX (1 μg/mL) and CIS (5 μg/mL) for 24 h. LDH assays revealed that U2OS cell cytotoxicity was remarkably higher in cells treated with a combination of PtNPs and DOX than in untreated cells or those treated with a single agent (Figure 6A). Similarly, Tanaka et al. [47] found that HSC-3-M3 cells treated with PtNCP beads at concentrations above 50 μg/mL exhibited greater LDH leakage than untreated cells and demonstrated increased extracellular LDH levels depending on the concentration of PtNCP beads. To further confirm the effect of PtNPs on membrane integrity and their cytotoxic effects against U2OS cells, PtNPs (10 μg/mL), DOX (1 μg/mL), or a combination of PtNPs (10 μg/mL) and DOX (1 μg/mL) or combination of CIS (5.0 μg/mL) and DOX (1 μg/mL) were incubated with the cells for 24 h and a trypan blue dye exclusion assay was performed. PtNPs (10 μg/mL) and DOX (1 μg/mL) significantly increased cell death via the loss of membrane integrity (Figure 6B). Similarly, the combination of CIS and DOX exhibited significant effect on cell membrane integrity. Although individual doses of both PtNPs and DOX increased LDH leakage and cell death, the effect was much more pronounced for the combined dose. Trypan blue assays have revealed that the combination of epigallocatechin-3-O-gallate (EGCG) and DOX promotes cell death and apoptosis in Hep3B cells by increasing the number of autophagic vesicles [51]. Moreover, DOX-conjugated magnetic NPs containing hydrolyzable hydrazone bonds coated with poly[*N*-(2-hydroxypropyl) methacrylamide] (PHPMA) exhibited enhanced cytotoxicity in both drug-sensitive and -resistant tumor cells compared to free DOX [52]. Al-Shakarchi et al. [53] reported that the combined use of clinical anticancer agents such as DOX, paclitaxel, oxaliplatin, vinblastine, and vincristine with cytochrome C-decorated hybrid NPs significantly increased apoptosis and cell death in HepG2, Huh-7D, and SK-hep-1 cells. Collectively, PtNPs and DOX impair membrane integrity and ultimately cause U2OS cell death.

### 3.9. PtNPs and DOX Alter Cell Morphology

**Figure 7:**
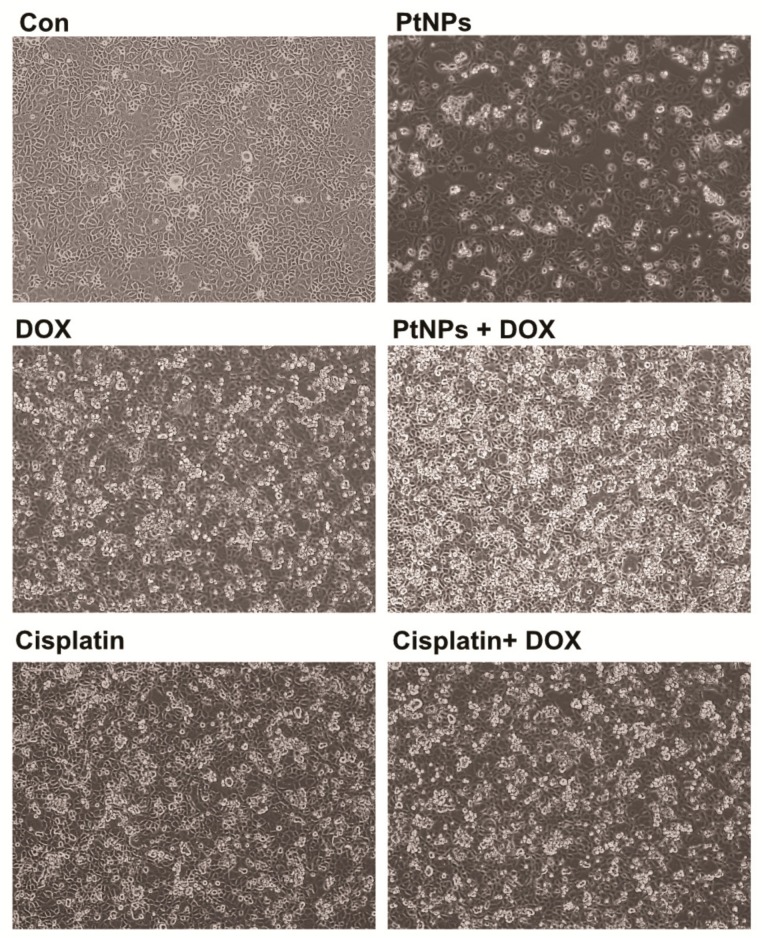
PtNPs and DOX alter U2OS cell morphology. The effect of PtNPs and DOX on cell morphology was determined using an optical microscope after exposure to PtNPs (10 μg/mL), DOX (1 μg/mL), CIS (5 μg/mL), or a combination of PtNPs (10 μg/mL) and DOX (1 μg/mL) or combination of DOX (1 μg/mL) and CIS (5 μg/mL) for 24 h. Results are expressed as the mean ± standard deviation of three independent experiments; at least three independent experiments were performed for each sample. Cells exposed to PtNPs and DOX had lost their typical shape and cell adhesion capacity and reduced in size and cell density. Scale bar: 200 µm.

To determine the impact of PtNPs and DOX on cell morphology, an optical microscope was used to monitor cell structure; untreated U2OS cells were compared with those treated with PtNPs (10 μg/mL), DOX (1 μg/mL), CIS (5 μg/mL), or a combination of PtNPs (10 μg/mL) and DOX (1 μg/mL) or combination of DOX (1 μg/mL) and CIS (5 μg/mL) for 24 h. The control cells were round and polygonal in shape and formed irregular confluent aggregates, whereas cells treated with either PtNPs or DOX had a narrow, spherical shape. Following the treatment of U2OS cells with PtNPs (10 μg/mL), DOX (1 μg/mL), or a combination of PtNPs (10 μg/mL) and DOX (1 μg/mL) for 24 h, obvious morphological changes, apoptotic features, and chromatin condensation were observed (Figure 7). The control cells appeared normal in shape and were attached to the surface, whereas those exposed to PtNPs and DOX had lost their typical shape and cell adhesion capacity and reduced in size and cell density. The combination of CIS and DOX exhibited a significant effect on cell morphology comparable with PtNPs and DOX. Although the individual doses of both PtNPs and DOX altered cell morphology and induced apoptosis, the effect was much more pronounced for the combined dose. Ortiz-Lazareno [54] observed that the combination of MG132 and DOX induced multilobular nuclei, increased cytoplasmic volume, and membrane blebbing in U937 leukemia cells, suggesting that U937 cells exhibit signs of morphological membrane damage and apoptosis. Our findings agree with this study and a previous report showing that AgNPs induce morphological changes in human cervical cancer cells [55]. These results clearly suggest that the toxic effects exerted by PtNPs and DOX-sensitize U2OS cells to the toxic effects of PtNPs and DOX, that the cytotoxic effect of PtNPs and DOX could be due to their antineoplastic nature and ability to induce cell death via numerous molecular mechanisms, and that the combination of PtNPs and DOX could induce U2OS cell apoptosis.

### 3.10. PtNPs and DOX Induce ROS Generation and Lipid Peroxidation in U2OS Cells

**Figure 8:**
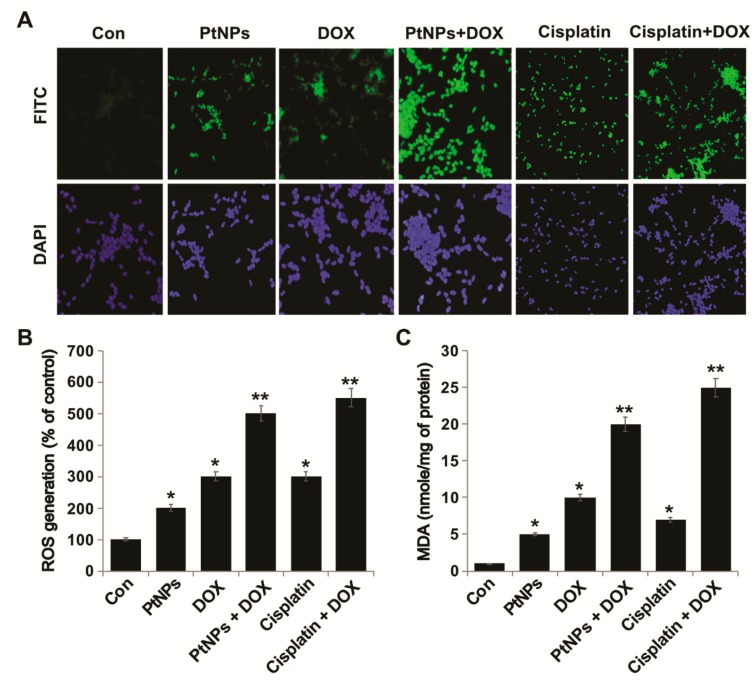
PtNPs and DOX induce ROS generation and lipid peroxidation in U2OS cells. U2OS cells were treated with PtNPs (10 μg/mL), DOX (1 μg/mL), CIS (5 μg/mL), or a combination of PtNPs (10 μg/mL) and DOX (1 μg/mL) or combination of DOX (1 μg/mL) and CIS (5 μg/mL) for 24 h. (**A**) ROS generation was measured using DCFH-DA-FITC with fluorescence microscopy. (**B**) Spectrophotometric ROS analysis was performed using DCFH-DA. (**C**) MDA concentrations were measured using a thiobarbituric acid-reactive substances assay and expressed as nanomoles per milliliter. Differences between the treated and control groups were measured using Student’s *t*-test and statistically significant differences are indicated by * (*p* < 0.05). The results are presented as mean ± standard deviation of three experiments. * *p* < 0.05 was considered significant; ** *p* < 0.01 was considered highly significant and *** *p* < 0.001 was considered very highly significant. Scale bar: 200 µm.

Metallic NPs such as Ag-, Au-, Pd-, and PtNPs are capable of inducing toxicity via ROS generation by interacting with cells, which is a feasible and reliable mechanism of toxicity [56,57]. ROS, such as the peroxide hydroxyl radical and singlet oxygen, play important roles in cell signaling and the homeostasis of biological processes. High levels of ROS in the body can result in oxidative damage to cellular constituents and cell metabolism dysfunction [58,59]; therefore, exogenous ROS generation is an effective therapeutic candidate for cancer therapy [60]. For example, AgNPs induce ROS generation in a variety of cancer cells, including human breast, lung, ovarian, and cervical cancer cells [20,57,61,62]. Furthermore, the combination of AgNPs with salinomycin [20] or gemcitabine [63] and the combination of PdNPs with tubastatin A increase the level of lipid peroxidation in human cancer cells [64]. Lycopene-reduced GOAgNP and PdNPs enhance the apoptotic potential of trichostatin A in human ovarian and cervical cancer cells, respectively [65,66]. To explore the potential of PtNPs and DOX to induce toxicity via ROS generation in U2OS cells, we measured ROS levels in cells treated with PtNPs (10 μg/mL), DOX (1 μg/mL), CIS (5 μg/mL), or a combination of PtNPs (10 μg/mL) and DOX (1 μg/mL) or combination of DOX (1 μg/mL) and CIS (5 μg/mL) for 24 h. To determine whether treatment with PtNPs and DOX would generate oxidative stress in U2OS cells, the cells were stained with DCFH-DA and ROS production was examined by measuring the proportion of cells positive for DCF-derived fluorescence after treatment. PtNPs and DOX stimulated ROS generation in U2OS cells (Figure 8A); The combination of CIS and DOX exhibited significant effect on ROS comparable with PtNPs and DOX although individual doses of both PtNPs and DOX increased ROS generation, the effect was much more pronounced for the combined dose. Higher levels of ROS were generated in cells treated with PtNPs and DOX than in untreated cells or those treated with either PtNPs or DOX alone. Similarly, the combination of PSHA-DOXNPs and Se-CurNPs was shown to increase cellular ROS generation to a greater extent than individual doses of either Se-CurNPs or PSHADOXNPs [45].

As shown in Figure 8B, PtNPs (10 μg/mL), DOX (1 μg/mL), or a combination of PtNPs (10 μg/mL) and DOX (1 μg/mL) increased ROS generation by 200, 300, and 500%, respectively, compared to the untreated control. These results suggest that the combination of PtNPs and DOX increases ROS generation more than DOX or PtNPs alone. NPs biologically synthesized using *Azadirachta indica* leaf extract were shown to induce ROS generation and cell death in HEK293 cells [67]. Moreover, DOX-induced apoptosis has been linked to ROS formation due to the redox activation of DOX [68]. Similarly, the combination of metallic NPs (such as AgNPs) with salinomycin increased ROS generation in human ovarian cancer cells [20], whilst AgNPs potentiated the apoptotic potential of gemcitabine via ROS generation in human ovarian cancer cells [63]. Other metallic NPs, such as PdNPs, can increase ROS generation due to tubastatin A in human breast cancer cells [64]. Apigenin-functionalized PtNPs and vanillin-functionalized GOPtNPs were shown to increase ROS generation in human monocytic THP-1 and prostate cancer (LNCaP) cells, respectively [23,46]. DOX and Pt drugs can activate nicotinamide adenine dinucleotide phosphate oxidases to generate superoxide radicals, prolonging their blood circulation and tumor accumulation and effectively inhibiting tumor growth and reducing the side effects of anticancer drugs [60].

Increased ROS generation may affect lipid peroxidation due to high levels of oxidative stress; therefore, we measured the levels of MDA, a lipid peroxidation biomarker. Exposure to PtNPs and DOX for 24 h induced lipid peroxidation in U2OS cells (Figure 8C) and increased their MDA levels in a statistically significant manner. PtNPs (10 μg/mL), DOX (1 μg/mL), or the combination of PtNPs (10 μg/mL) and DOX (1 μg/mL) increased MDA levels by 5, 10, and 20%, respectively (Figure 8C). Although individual doses of both PtNPs and DOX increased MDA levels, the effect was much more pronounced for the combined dose. The combination of CIS and DOX exhibited significant effect on MDA production which is comparable with PtNPs and DOX. Metallic NPs, such as AgNPs, have been shown to increase MDA levels in variety of cancer cells, including human breast, lung, ovarian, and cervical cancer cells [57,61,62]. Furthermore, the combination of AgNPs with salinomycin [20] or gemcitabine [63] and the combination of PdNPs with tubastatin A increased lipid peroxidation levels in human cancer cells [64]. Oxidative stress is known to be involved both NP- and chemotherapeutic drug-induced cell death; in this study, increases in lipid peroxidation levels suggested that oxidative stress has a role in the death of U2OS cells exposed to PtNPs and DOX. Recently, Almeer et al. [67] reported that PtNPs increased lipid peroxidation by increasing MDA levels in HEK293 cells. Our results clearly suggest that increases in ROS generation and lipid peroxidation could be possible mechanisms of PtNP- and DOX-induced toxicity.

### 3.11. PtNPs and DOX Increase the Levels of NO and Carbonylated Protein 

**Figure 9:**
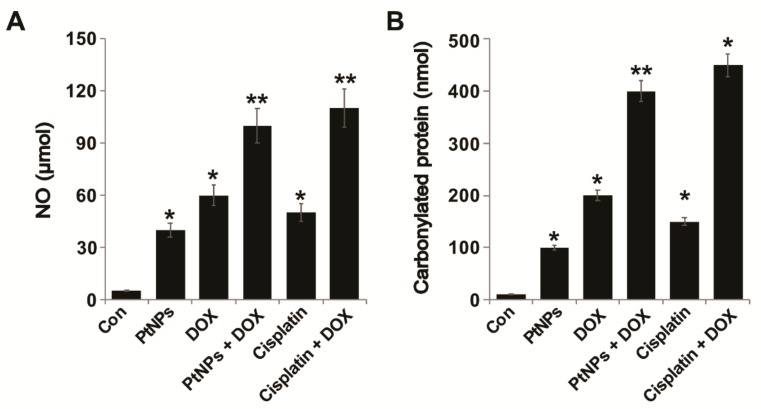
PtNPs and DOX increase the NO and protein carbonyl content of U2OS cells. U2OS cells were treated with PtNPs (10 μg/mL), DOX (1 μg/mL), CIS (5 μg/mL), or a combination of PtNPs (10 μg/mL) and DOX (1 μg/mL) or combination of DOX (1 μg/mL) and CIS (5 μg/mL) for 24 h. (**A**) NO production was quantified spectrophotometrically using Griess reagent and expressed as micromoles. (**B**) Protein carbonyl content was measured and expressed as nanomoles. Differences between the treated and control groups were measured using Student’s *t*-test and statistically significant differences are indicated by * (*p* < 0.05). The results are presented as mean ± standard deviation of three experiments. * *p* < 0.05 was considered significant; ** *p* < 0.01 was considered highly significant and *** *p* < 0.001 was considered very highly significant.

NO is an endogenous bioregulator that plays various critical pathophysiological roles. In tumor cells, NO levels play a vital role in proliferation; for instance, low NO levels are unable to control the proliferation, survival, and apoptosis of tumor cells [69]. Maximizing NO levels in cancer cells is necessary to obtain satisfactory therapeutic effects; therefore, we examined whether the combination of PtNPs and DOX could increase NO levels in U2OS cells. U2OS cells were treated with PtNPs (10 μg/mL), DOX (1 μg/mL), CIS (5 μg/mL), or a combination of PtNPs (10 μg/mL) and DOX (1 μg/mL) or combination of DOX (1 μg/mL) and CIS (5 μg/mL) for 24 h and then NO levels were measured. As shown in Figure 9A, incubation with PtNPs DOX and a combination of PtNPs and DOX had a significant effect compared with the control. As expected, treating cells with PtNPs and DOX caused a remarkable level of NO which correlated with reduced viability, indicating the significant cytotoxic effect of PtNPs and DOX. The combination of CIS and DOX exhibited significant effect on NO and carbonylated protein contents. Although individual doses of both PtNPs and DOX increased NO levels, the effect was much more pronounced for the combined dose. Recently, Cao et al. [70] demonstrated that PtNPs triggered a nine- to ten-fold increase in NO release from donors such as S-nitroso-N-acetylpenicillamine (SNAP) and S-nitrosoglutathione (GSNO), which is major cause of cytotoxic effects in A549 cells. Apigenin-functionalized PtNPs and vanillin-functionalized GOPtNPs increased NO levels in human monocytic THP-1 and prostate cancer (LNCaP) cells, respectively [23,46]. Collectively, these findings demonstrate that PtNPs and DOX triggered NO release, efficiently killing cancer cells.

ROS production is balanced by the detoxification of radical species by antioxidants and is involved in various cellular signaling pathways. Increased ROS levels can damage cellular components such as proteins, lipids, and DNA [71,72,73], whilst prolonged oxidative stress can induce apoptosis and cell death. The effect of PtNPs and DOX on the fate of carbonylated proteins in tumor cells has not yet been investigated; therefore, we measured the carbonyl protein content of PtNP- and DOX-treated U2OS cells in order to better understand their mechanisms of cytotoxicity. The cells were treated with PtNPs (10 μg/mL), DOX (1 μg/mL), CIS (5 μg/mL), or a combination of PtNPs (10 μg/mL) and DOX (1 μg/mL) or combination of DOX (1 μg/mL) and CIS (5 μg/mL) for 24 h and then carbonylated protein content was measured. As shown in Figure 9B, PtNPs, DOX, and the combination of PtNPs and DOX significantly increased carbonyl protein content by 100, 200, and 400 nM, respectively, compared with control. Although individual doses of both PtNPs and DOX increased the carbonylated protein content, the effect was much more pronounced for the combined dose. As expected, treating cells with PtNPs and DOX significantly increased carbonylated protein content, which is correlated with reduced viability, and significantly increased the cytotoxic effect of PtNPs and DOX. The combination of CIS and DOX significantly increased the level of ROS, MDA, NO, and carbonylated protein production, which is comparable with PtNPs and DOX. These results confirmed that the combination of PtNPs and DOX might cause the accumulation of carbonylated proteins, a major cause of cytotoxicity. A previous study demonstrated that combining photodynamic therapy (PDT) with proteasome inhibitors caused the accumulation of carbonylated proteins in the endoplasmic reticulum (ER), aggravated ER stress, and potentiated tumor cell cytotoxicity [74]. Similarly, CeO_2_NPs and DOX increased carbonyl protein levels in the human malignant melanoma cell line A375 [41]. Previously, we reported that PtNPs and vanillin-functionalized GOPtNPs increased the carbonylated protein content of human monocytic THP-1 and prostate cancer (LNCaP) cells, respectively [23,46]. Collectively, these data suggest that increased carbonylated protein levels are a major cause of cytotoxicity.

### 3.12. Effect of PtNPs and DOX on Antioxidants

**Figure 10:**
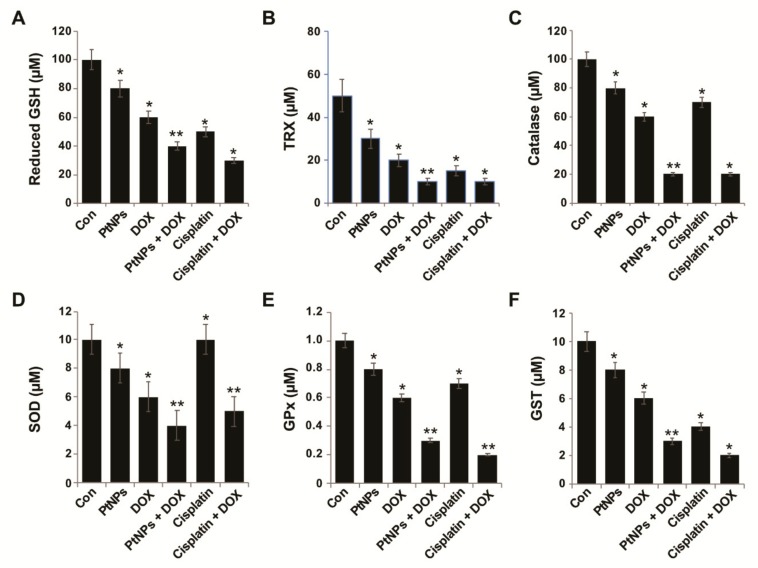
Effect of PtNPs and DOX on antioxidant markers. U2OS cells were treated with PtNPs (10 μg/mL), DOX (1 μg/mL), CIS (5 μg/mL), or a combination of PtNPs (10 μg/mL) and DOX (1 μg/mL) or combination of DOX (1 μg/mL) and CIS (5 μg/mL) for 24 h. After incubation, the cells were harvested, washed twice with an ice-cold PBS solution, collected, and disrupted by ultrasonication for 5 min on ice. (**A**) Glutathione reductase activity (GSH) was measured by monitoring the rate of NADPH oxidation. Oxidation of NADPH was monitored spectrophotometrically in kinetic mode for 5 min at 340 nm. Glutathione reductase activity was proportional to the rate of absorbance decrease. (**B**) Thioredoxin reductase (TRX) activity was measured by monitoring the conversion of DTNB to TNB by reduced thiols. Cell lysate was mixed with 100 μL of solution containing 5 mM DTNB and 250 μM NADPH. Absorbance was monitored spectrophotometrically in kinetic mode for 5 min at 405 nm. Thioredoxin reductase activity was proportional to the difference in TNB generation rate in samples. (**C**) Catalase activity was assayed by monitoring the rate of removal of exogenously added hydrogen peroxide in a colorimetric reaction. Absorbance was measured at 520 nm. Catalase activity was proportional to the difference in absorbance between a control sample and treated sample. (**D**) Superoxide dismutase activity was assayed by monitoring the rate of removal of exogenously added superoxide. Superoxide dismutase activity was proportional to the difference in absorbance increase rate between a control sample and the treated sample. (**E**) Glutathione peroxidase activity (GPx) was measured indirectly by monitoring NADPH consumption in a coupled reaction with glutathione reductase and (**F**) Glutathione S-Transferase (GSTs) activity was assayed spectrophotometrically at 25 °C with reduced glutathione (GSH) and 1-chloro-2, 4-dinitrobenzene (CDNB) as substrates. GST concentrations are expressed as micromoles. Differences between the treated and control groups were measured using Student’s *t*-test and statistically significant differences are indicated by * (*p* < 0.05). The results are presented as mean ± standard deviation of three experiments. * *p* < 0.05 was considered significant; ** *p* < 0.01 was considered highly significant and *** *p* < 0.001 was considered very highly significant.

The intracellular thiol redox state is an endogenous effector involved in regulating the mitochondrial permeability transition pore complex and is a major causal factor of mitochondrion-based cell death, with GSH depletion causing apoptosis [75]. Cells possess various antioxidant systems, including superoxide dismutase, catalase, GSH, thioredoxins, glutathione peroxidase, and glutathione S-transferases [75]. The downregulation of these antioxidant defenses can increase ROS levels, alter redox status, and cause cellular damage. Thus, we measured the levels of all these antioxidants in U2OS cells treated with PtNPs (10 μg/mL), DOX (1 μg/mL), CIS (5 μg/mL), or a combination of PtNPs (10 μg/mL) and DOX (1 μg/mL) or combination of DOX (1 μg/mL) and CIS (5 μg/mL) for 24 h. PtNPs and DOX significantly decreased the levels of all antioxidants by at least five-fold compared to the untreated cells (Figure 10A–F). The combination of CIS and DOX significantly decreased the level of antioxidants, which is comparable with PtNPs and DOX. GSH plays an important role in eliminating reactive oxygen species (ROS) and regulating various genes involved in cell death [76]. Cys residue oxidation modulates various receptor tyrosine kinases, including the MAPKs, ERK, p38, and JNK, which are central players in stress-induced apoptosis mechanisms [77]. When oxidative stress levels are high, glutaredoxin and thioredoxin induce apoptosis signal-regulating kinase involved in apoptosis initiation [78]. Metallic NPs, such as AgNPs, have been shown to reduce antioxidant levels in a variety of cancer cells including human breast, lung, ovarian, and cervical cancer cells [57,61,62]. Furthermore, combining AgNPs with salinomycin [20] or gemcitabine [63] and combining PdNPs with tubastatin A decreased the antioxidant level in human cancer cells. Recently, we reported that PtNPs and GOPtNPs reduce the levels of various antioxidants in human monocytic THP-1 and human prostate cancer (LNCaP) cells, respectively [23,46]. When Wistar rats were exposed to three forms of DOX (conventional (DOX), liposome-encapsulated DOX (lipoDOX), and apoferritin-encapsulated DOX (apoDOX)), conventional DOX was shown to reduce the plasma levels of 4-hydroxy-2-nonenal (4-HNE) and thioredoxin reductase 2 (TXNRD2) [79]. Thioredoxins play important roles in various physiological processes, such as proliferation, angiogenesis, and metastasis, are redox regulators of a number of transcription factors (NF-ĸB, HIF1-α, and VEGF), and modulate matrix metalloproteinase-9 (MMP-9) [80]. Catalase is a vital antioxidant which facilitates hydrogen peroxide neutralization, prevents mutagenesis, and protect the cells from oxidative damage; however, PtNPs and DOX decreased catalase levels and thus increased apoptosis via H_2_O_2_. Decreased antioxidant levels impair the cellular redox balance and eventually lead to cell death.

### 3.13. PtNPs and DOX Reduce the Mitochondrial Membrane Potential (MMP) and ATP Levels

**Figure 11:**
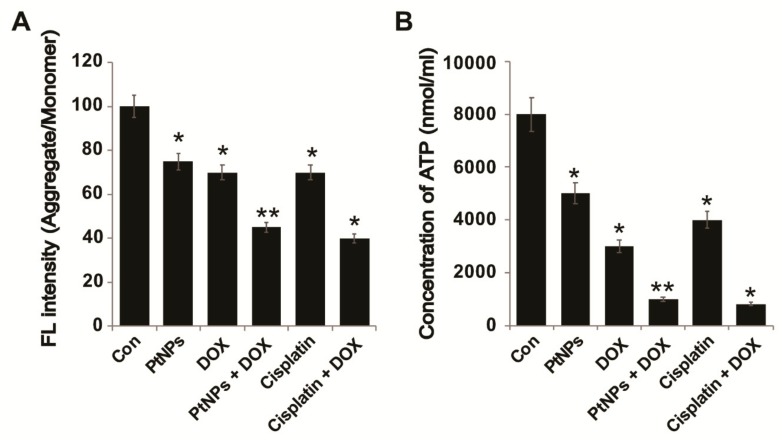
PtNPs and DOX decrease the MMP and ATP content. U2OS cells were treated with PtNPs (10 μg/mL), DOX (1 μg/mL), CIS (5 μg/mL), or a combination of PtNPs (10 μg/mL) and DOX (1 μg/mL) or combination of DOX (1 μg/mL) and CIS (5 μg/mL) for 24 h and: (**A**) the MMP was determined using JC-1, a cationic fluorescent indicator; the (**B**) intracellular ATP content was determined according to the manufacturer’s instructions (Sigma-Aldrich, St. Louis, MO, USA; Catalog Number MAK135). Differences between the treated and control groups were measured using Student’s *t*-test and statistically significant differences are indicated by * (*p* < 0.05). The results are presented as mean ± standard deviation of three experiments. * *p* < 0.05 was considered significant; ** *p* < 0.01 was considered highly significant and *** *p* < 0.001 was considered very highly significant.

To determine the MMP (ΔΨm) in U2OS cells, they were treated with PtNPs (10 μg/mL), DOX (1 μg/mL), CIS (5 μg/mL), or a combination of PtNPs (10 μg/mL) and DOX (1 μg/mL) or combination of DOX (1 μg/mL) and CIS (5 μg/mL) for 24 h; the results are presented in Figure 11A. The MMP changed significantly in all treated U2OS cells, with the combination of PtNPs and DOX inducing remarkable membrane potential loss. Individually, PtNPs and DOX caused loss of membrane potential in 30 ± 3.5 and 25 ± 2.5% cells, respectively, with the combination of PtNPs and DOX inducing a greater ΔΨm loss in 50 ± 5.9% U2OS cells compared with the remaining groups (*p* < 0.05). Similarly, the combination of CIS and DOX significantly decrease the level of MMP and ATP which are critical factors for maintaining cell viability. Ortiz-Lazareno et al. [54] reported that the combination of MG132 and DOX induced significant MMP loss in U937 leukemia cells, whilst metallic NPs, such as AgNPs, have been shown to alter the MMP in variety of cancer cells, including human breast, lung, ovarian, and cervical cancer cells [57,61,62]. Furthermore, combining AgNPs with salinomycin [20] or gemcitabine [63] and combining PdNPs with tubastatin A decreased the MMP in human cancer cells [64]. When human dermal fibroblasts were treated with AgNPs, they internalized into the mitochondrial outer membrane, directly causing mitochondrial damage and disturbing respiratory chain function by increasing ROS levels and oxidative stress. Conversely, the nanosized dendrimer polyamidoamine (PAMAM; average size, 45 nm) was found to colocalize with mitochondria, eventually disrupting the MMP, causing the release of cytochrome c, and activating caspases 3 and 9 [81]. High levels of oxidative stress lead to increased ROS levels, which cause mitochondrial membrane damage and induce toxicity [25,82]. Kumari et al. [45] reported that the combination of PSHA-DOXNPs and Se-CurNPs decreased the MMP of cells to a greater extent than individual doses of Se-CurNPs and PSHA-DOXNPs. Recently, we reported that PtNPs and GOPtNPs decreased the MMP in human monocytic THP-1 and prostate cancer (LNCaP) cells, respectively [23,46]. These results indicate that the loss of MMP directly correlates with ROS generation and oxidative stress in PtNP- and DOX-induced U2OS cell death due to free radical generation.

Mitochondria play a critical role in energy supply and apoptosis execution. DOX can increase oxidative stress by interacting with respiratory chain complex I, which can alter the MMP, increase mitochondrial permeability, and reduce ATP levels [83,84,85]. ATP production and ROS generation by mitochondria are involved in various physiological processes. Furthermore, mitochondria are crucial regulators of intracellular Ca^2+^ levels and ROS homeostasis which are vital for cell viability, signaling, differentiation, death, maintaining control over the cell cycle, and cell growth [86]. As a result, MMP loss can decrease the proton gradient that drives H^+^ through ATP synthase, reducing mitochondrial ATP production. Therefore, we examined the level of ATP in cells treated with PtNPs (10 μg/mL), DOX (1 μg/mL), CIS (5 μg/mL), or a combination of PtNPs (10 μg/mL) and DOX (1 μg/mL) or combination of DOX (1 μg/mL) and CIS (5 μg/mL) for 24 h. ATP production was significantly affected in all tested samples, with ATP production dramatically lower in cells treated with PtNPs and DOX (Figure 11B). Metallic NPs such as AgNPs can cause mitochondrial depolarization accompanied by reduced ATP production in A549 cells [87]. These results indicate that the combination of PtNPs and DOX could increase mitochondrial membrane permeabilization, leading to mitochondrial damage, reduced MMP, altered permeability, the release of death-related molecular signals, increased oxidative stress, and depleted ATP, causing cell death.

### 3.14. PtNPs and DOX Enhance Proapoptotic and Downregulate Antiapoptotic Gene Expression

**Figure 12:**
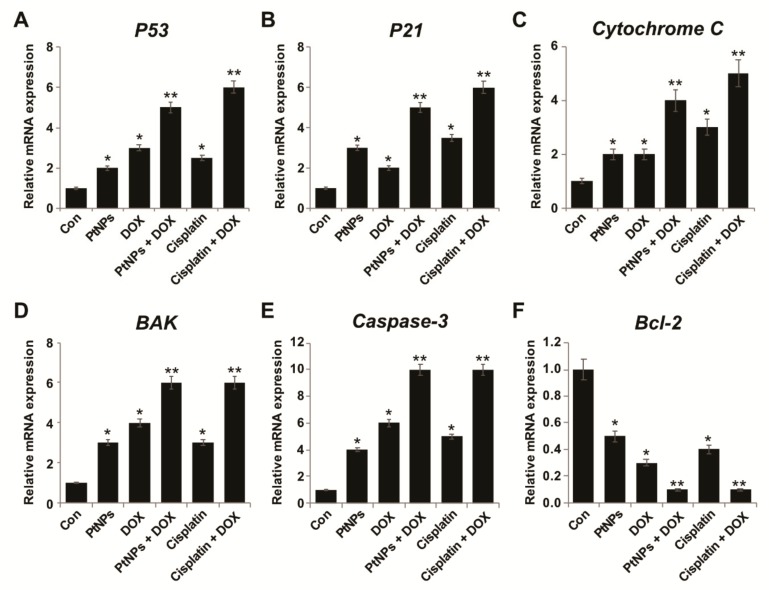
Effect of PtNPs and DOX on expression of pro and antiapoptotic genes (**A**–**F**). U2OS cells were treated with PtNPs (10 μg/mL), DOX (1 μg/mL), CIS (5 μg/mL), or a combination of PtNPs (10 μg/mL) and DOX (1 μg/mL) or combination of DOX (1 μg/mL) and CIS (5 μg/mL) for 24 h and their relative mRNA expression of apoptotic and antiapoptotic genes was analyzed by quantitative RT-PCR. Expression was determined as the fold change compared to GAPDH expression. Differences between the treated and control groups were measured using Student’s *t*-test and statistically significant differences are indicated by * (*p* < 0.05). The results are presented as mean ± standard deviation of three experiments. * *p* < 0.05 was considered significant; ** *p* < 0.01 was considered highly significant and *** *p* < 0.001 was considered very highly significant.

Oxidative stress-induced changes in the intracellular redox status are important for altering apoptotic and antiapoptotic gene expression; therefore, we examined how PtNP- and DOX-induced oxidative stress might influence gene expression. We employed RT-PCR to determine the mRNA expression of the apoptosis-associated proteins p53, p21, Cyt C, Bak, caspase 3, and Bcl-2 in U2OS cells treated with PtNPs (10 μg/mL), DOX (1 μg/mL), CIS (5 μg/mL), or a combination of PtNPs (10 μg/mL) and DOX (1 μg/mL) or combination of DOX (1 μg/mL) and CIS (5 μg/mL) for 24 h. The p53, p21, Cyt C, Bak, and caspase 3 genes were upregulated in PtNP- and DOX-treated U2OS cells compared with untreated cells (*p* < 0.05), with the greatest upregulation observed for caspase-3. Likewise, PtNPs and DOX induced the downregulation of the Bcl-2 antiapoptotic gene (*p* < 0.05) compared with untreated cells. We also observed upregulation of the p53, p21, Cyt C, Bak, and caspase 3 genes when the cells were treated with either PtNPs or DOX alone; however, the effect was significantly greater and the highest upregulation was observed in cells treated with both PtNPs and DOX (*p* < 0.05; Figure 12A–F). The combination of CIS and DOX significantly decrease the level of antiapoptotic genes and increase apoptotic genes expression. Although individual doses of both PtNPs and DOX increased proapoptotic gene expression, the effect was much more pronounced for the combined dose. These data suggest that the treatment of U2OS cells with PtNPs and DOX favors the activation of proapoptotic genes. Gene expression analysis of apoptosis-associated proteins revealed that the combination of PtNPs and DOX upregulated the expression of p53, p21, Cyt C, Bak, and caspase 3 and downregulated Bcl-2 expression, indicating up to five-fold higher levels of apoptosis in the treated cells. Recently, Kumari et al. (2018) [45] reported that Se-CurNPs increase the anticancer potential of DOX by increasing the expression of the pro-apoptotic protein Bax, increasing Cyt C release, and downregulating Bcl-2. p53 is a tumor suppressor gene capable of inducing cell cycle arrest and apoptosis [88]. Bax is a proapoptotic protein that forms pores in the mitochondrial membrane through which Cyt C is released, further activating the downstream apoptotic pathway. DOX has been reported to induce apoptosis via Bax overexpression [89,90], consistent with our findings. Bcl-2 is an antiapoptotic protein that plays a major role in cell survival and inhibits the action of proapoptotic proteins. Bcl-2 was downregulated nine-fold by the combination of PtNPs and DOX, whereas PtNPs and DOX each caused a five-fold downregulation and seven-fold upregulation in Bcl-2 expression, respectively (Figure 12A–F). Furthermore, the data obtained from the gene expression analysis of Cyt C and caspase 3 delineates the mechanism of apoptosis. Increased Cyt C levels enhanced the release of Cyt C from the mitochondria in cells treated with both PtNPs and DOX, thus might be a consequence of the synergistic action of the PtNPs and DOX on apoptosis induction. Caspase activation depends on the release of Cyt C from the mitochondria and leads to caspase-mediated apoptosis [91]. Collectively, the gene expression analysis of key apoptotic genes indicated that combining PtNPs and DOX increased apoptosis-mediated cell death in U2OS cells.

### 3.15. PtNPs and DOX Increase 8-oxo-dG and oxo-G Levels

**Figure 13:**
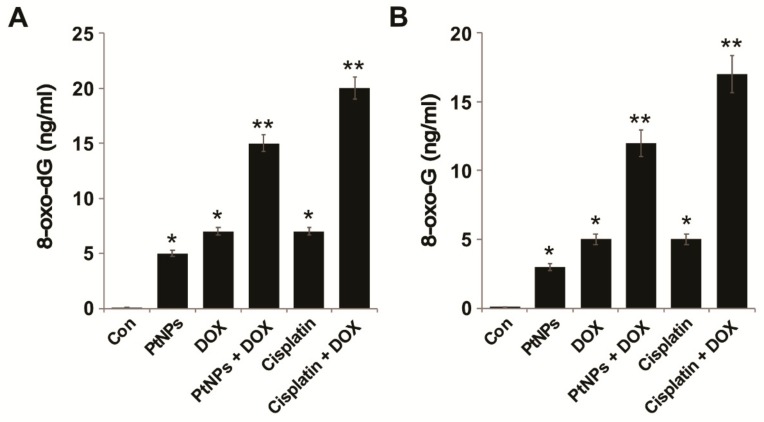
PtNPs and DOX increase oxidative damage to DNA and RNA. U2OS cells were treated with PtNPs (10 μg/mL), DOX (1 μg/mL), CIS (5 μg/mL), or a combination of PtNPs (10 μg/mL) and DOX (1 μg/mL) or combination of DOX (1 μg/mL) and CIS (5 μg/mL) for 24 h and then (**A**) 8-oxo-dG and (**B**) 8-oxo-G levels were measured. Differences between the treated and control groups were measured using Student’s *t*-test and statistically significant differences are indicated by * (*p* < 0.05). The results are presented as mean ± standard deviation of three experiments. * *p* < 0.05 was considered significant; ** *p* < 0.01 was considered highly significant and *** *p* < 0.001 was considered very highly significant.

Oxidative stress allows cells to maintain their physiological status; it can oxidize carbohydrates, lipids, proteins, and DNA, leading to cellular dysfunction and ultimately cell death by inducing single- and double-strand DNA fragmentation [92]. The oxidation products 8-oxo-dG and 8-oxo-G are abundant lesions in genomic, mitochondrial, and telomeric DNA and RNA and are potential markers of oxidative stress that preferentially accumulate at the 5’ end of guanine strings in the DNA helix, guanine quadruplexes, and RNA molecules [93]. Although there are various oxidative nucleic acid damage products, 8-oxo-dG and 8-oxo-G are suitable biomarkers of oxidative damage to DNA and RNA, respectively. We measured the levels of 8-oxo-dG and 8-oxo-G in U2OS cells exposed PtNPs (10 μg/mL), DOX (1 μg/mL), CIS (5 μg/mL), or a combination of PtNPs (10 μg/mL) and DOX (1 μg/mL) or combination of DOX (1 μg/mL) and CIS (5 μg/mL) for 24 h. The levels of 8-oxo-dG and 8-oxo-G were significantly higher in cells exposed to both PtNPs and DOX; The combination of CIS and DOX significantly increase the level of 8-oxo-dG and 8-oxo-G levels, which is comparable with PtNPs and DOX. Although individual doses of PtNPs and DOX increased 8-oxo-dG and 8-oxo-G levels, the effect was much more pronounced for the combined dose (Figure 13A,B). Oxidative stress-induced DNA damage has significant effects on proliferation, differentiation, and cell-to-cell signaling [94]. It is well known that NP-induced ROS could generate 8-oxo-7, 8-dihydro-2′-deoxyguanosine (8-oxodG), and 8-oxo-G, markers of oxidative DNA and RNA damage [95]. 8-oxo-dG and 8-oxo-G are the most predominant ROS-induced oxidative modifiers among the different oxidative products [96,97], the most abundant oxidized products, stable, and are relatively easily formed. Carbon nanomaterials, such as GO and reduced GO, have been shown to cause oxidative DNA damage and increase 8-oxo-dG and 8-oxo-G levels in THP-1 cells [98], whilst PtNPs and GOPtNPs increased 8-oxo-dG and 8-oxo-G levels in human monocytic THP-1 and prostate cancer (LNCaP) cells, respectively [23,46]. It is possible that DNA damage is induced by PtNPs and DOX entering the nucleus and binding DNA [99]. Recently, Wang et al. (2018) [100] reported that the combination of genistein (GEN) and DOX-loaded polypeptide NPs (DOX-NPs) significantly increased oxidative stress and DNA damage by increasing 8-oxo-G levels in prostate cancer cells; consequently, the coadministration of GEN and DOX-NPs triggered more apoptosis and produced more DNA breaks. Collectively, these findings confirmed that 8-oxo-dG levels are positively correlated with oxidative damage levels and could be a mechanism via which PtNPs and DOX directly induce DNA damage in U2OS cells.

### 3.16. PtNPs and DOX Alter DNA Repair Gene Expression

**Figure 14:**
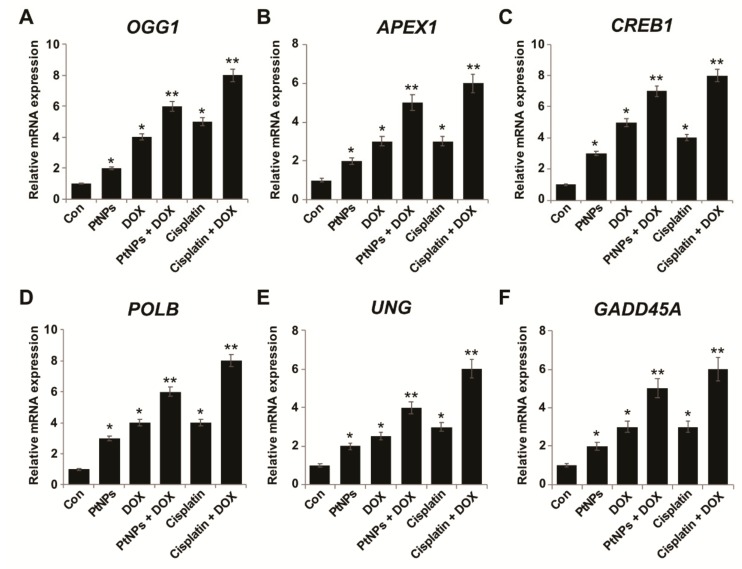
PtNPs aberrantly upregulate DNA damage and repair genes (**A**–**F**). U2OS cells were treated with PtNPs (10 μg/mL), DOX (1 μg/mL), CIS (5 μg/mL), or a combination of PtNPs (10 μg/mL) and DOX (1 μg/mL) or combination of DOX (1 μg/mL) and CIS (5 μg/mL) for 24 h and the relative mRNA expression of DNA damage genes was analyzed by quantitative RT-PCR. Expression was determined as the fold change compared to GAPDH expression. Differences between the treated and control groups were measured using Student’s *t*-test and statistically significant differences are indicated by * (*p* < 0.05). The results are presented as mean ± standard deviation of three experiments. * *p* < 0.05 was considered significant; ** *p* < 0.01 was considered highly significant and *** *p* < 0.001 was considered very highly significant.

DOX is known to intercalate DNA and inhibit DNA replication, whilst NPs can induce DNA damage. DNA lesions are predominantly repaired by the base excision repair (BER) gene expression pathway. We investigated the combined effects of PtNPs and DOX on the sequential expression of various genes involved in DNA repair in U2OS cells in order to understand whether DNA repair is an adoptive response against PtNP- and DOX-induced genotoxicity. U2OS cells were treated with PtNPs (10 μg/mL), DOX (1 μg/mL), CIS (5 μg/mL), or a combination of PtNPs (10 μg/mL) and DOX (1 μg/mL) or combination of DOX (1 μg/mL) and CIS (5 μg/mL) for 24 h and RT-PCR was used to determine the pattern of gene expression. We determined the expression levels of DNA glycosylases involved in BER, including *OGG1*, *APEX1*, *CREB1*, *POLB*, *UNG*, and *GADD45A*. Combination of CIS and DOX increase the expression of all tested genes. Although individual doses of both PtNPs and DOX increased DNA repair gene expression, the effect was much more pronounced for the combined dose (Figure 14A–F). 8-Oxoguanine DNA glycosylase 1 (OGG1) is a BER enzyme that removes oxidatively-damaged guanine from double stranded DNA [101,102]. A previous study reported the upregulation of the DNA damage genes OGG1 and APE1 in C57BL/6J mice treated with engineered NPs [103]. DNA damage-induced 45α (GADD45α) has been found to promote DNA repair and remove methylation markers involved in genomic stability. GADD45α mRNA levels are significantly higher in tumor tissue than in adjacent normal tissue [103]. GO and reduced GO cause oxidative DNA damage by increasing 8-oxo-dG and 8-oxo-G levels in THP-1 cells [98], whilst PtNPs and GOPtNPs increase DNA repair gene expression in human monocytic THP-1 and prostate cancer (LNCaP) cells, respectively [23,46]. Hepatocellular carcinoma cells (HepG2) treated with TiO2 NPs exhibited oxidative stress and ROS generation, which caused double strand breaks (DSBs), chromatin condensation, nuclear fragmentation, and apoptosis [104]. Metallic NPs, such as AgNPs, have been shown to induce 8-oxoG, DSBs, chromosomal damage, and DNA deletions in wild type and Ogg1-deficient mice [105], whilst cells exposed to TiO_2_ exhibit DNA damage, genome rearrangements, single strand breaks, DSBs, and intra/interstrand breaks [106,107]. The upregulation of BER pathway genes is a result of the genotoxic properties of PtNPs and DOX. Altogether, these findings suggest that U2OS cells are sensitive to the DNA-damaging combination therapy.

## 4. Conclusions

Recently, NP-mediated combination chemotherapies have received tremendous attention; therefore, it is necessary to identify suitable nontoxic and biocompatible NPs that can increase the therapeutic index of clinical anticancer drugs. In this study, PtNPs (10 μg/mL) were found to have a positive pharmacological interaction even with low doses of DOX (1.0 μg/mL) that are more tolerable, associated with fewer undesirable side effects, and have higher efficacy. This study also demonstrated that PtNPs could supplement conventional chemotherapeutic drugs, such as DOX. The combination of PtNPs and DOX exhibited considerable anticancer activity in U2OS cells by significantly reducing cell viability and proliferation and altering cell morphology. We observed synergistic effects on U2OS cell cytotoxicity, with increased LDH leakage, reactive oxygen species generation, and increased levels of malondialdehyde, nitric oxide, and carbonylated proteins. Mitochondrial dysfunction was confirmed by the loss of MMP, decreased ATP levels, and the upregulation of apoptotic and downregulation of antiapoptotic genes. Oxidative stress was confirmed as a major cause of cytotoxicity and genotoxicity since the levels of various antioxidants were decreased. Furthermore, PtNPs and DOX increased the levels of 8-oxo-dG and 8-oxo-G and induced DNA damage repair gene expression. Importantly, PtNPs increased oxidative stress and DNA damage in OS; therefore, the application of PtNPs in conjunction with DOX can be considered a desirable strategy. However, further studies are warranted to decipher the molecular mechanisms of the combination therapy for use against OS. Collectively, our data suggest that the combination of PtNPs and DOX could be a potent anticancer therapeutic strategy for OS and could contribute towards the development of novel modalities for therapeutic OS intervention.

## Figures and Tables

**Table 1:** List of primers used for quantitative real-time polymerase chain reaction for analysis of apoptotic and DNA damage gene expression.

Gene	List of Primers
**APEX1**	F:ATTGGCTGGAGGGCAGATCT
	R:CCACTGGGTGAGGTTTTCTGA
**OGG1**	F:TCCTCCCTAGGTTTCCTCTC
	R:TGAGACTAGTGACAGTGTTGG
**P53**	F:AGAGACCGTACAGAAGA
	R:CTGTAGCATGGGATCCTTT
**P21**	F:GTTGCTGTCCGGACTACCG
	R:AAAAACAATGCCACCACTCC
**Caspase-3**	F:AGGGGTCATTTATGGGACA
	R:TACACGGGATCTGTTTCTTTG
**Cyt C**	F: GCGTGTCCTTGGACTTAGAG
	R: GGCGGCTGTGTAAGAGTATC
**Bax**	F:CGAGCTGATCAGAACCATCA
	R:GAAAAATGCCTTTCCCCTTC
**POLB**	F:GTTTCAGAAGAGGTGCAGAG
	R:AGTGAAATAGAGAACACCACAG
**Bcl-2**	F:TAAGCTGTCACAGAGGGGCT
	R:TGAAGAGTTCCTCCACCACC
**CREB1**	F:CAGTTCAGTCTTCCTGTAAGGACT
	R:CGTTTGTCATGGTTAGTGTC
**UNG**	F:CTCTGCTTTAGTGTTCAAAGG
	R:GAGTTCTGATTTAGCCAGGA
	R:CCTTTGTACCGTTGCATCCT
**GAPDH**	F:AGGTCGGTGTGAACGGATTTG
	R:TGTAGACCATGTAGTTGAGGTCA
